# Physicochemical properties, microstructures, nutritional components, and free amino acids of *Pleurotus eryngii* as affected by different drying methods

**DOI:** 10.1038/s41598-019-56901-1

**Published:** 2020-01-10

**Authors:** Rui-Lin Yang, Qin Li, Qing-Ping Hu

**Affiliations:** 1 Analysis and Test Center, Shanxi Normal University, Linfen City, 041004 China; 2 School of Food Science, Shanxi Normal University, Linfen City, 041004 China

**Keywords:** Carbohydrates, Biochemical assays

## Abstract

In this study, we determined the influences of different drying techniques such as natural air (ND), hot-air (HD), vacuum (VD), infrared (ID), microwave (MD), and freeze drying (FD) methods on the color, shrinkage ratio (SR), rehydration ratio (RR), firmness, crispness, microstructures, nutritional components, and free amino acids of *Pleurotus eryngii*. The results showed that these parameters were markedly influenced by different drying techniques. Among them, FD was the most effective drying method which retained the main characteristics of the fresh *P. eryngii* in above mentioned indexes, followed by ND and HD at 40 °C. Finally, despite the least drying time, MD treatment was not suitable to the drying process of *P. eryngii* slices since it damaged physicochemical properties and caused massive losses of the main nutrients and free amino acids. The results will provide a theoretical basis for industrial processing of *P. eryngii*.

## Introduction

*Pleurotus eryngii*, often referred to as the king oyster mushroom, is called Xingbaogu in China. It is one of the important species of edible mushrooms commonly and has been widely cultivated and consumed in China because of its delicious taste, remarkable nutritional values and biological activities including antioxidant, antitumor, immunostimulatory activities, and modulating human gut microbiome^1–3^. The *P. eryngii* can be widely cultivated in lots of agricultural and industrial wastes conveniently and cheaply^4^. The production of the cultivated *P. eryngii* increased substantially at a rate of about 3 × 10^8^ kg/year in Asia^5^. Fresh *P. eryngii* slices can be cooked and retained its own firm textures^1^, and also can be fabricated into canned foods. Nevertheless, fresh *P. eryngii* is also one of the most perishable food raw materials and readily loses the best edible quality immediately after harvest because of its high in respiration rate, moisture content and nutrients, all of which made it easy to lose water or be attacked by microbes. Its shelf-life only reached about 3 weeks under the appropriate conditions^6,7^. Therefore, an effective preservation method was required for the industrial production and application of *P. eryngii* to extend its shelf-life.

Drying is a widely used and comparatively cheaper method for shelf-life extension of highly perishable mushrooms as it limited microbial growth, inhibited enzyme activity, and slowed down many moisture mediated reactions^8–10^. In addition, dried *P. eryngii* can be also accepted by consumers with a similar high scores like fresh *P. eryngii* in all acceptability attributes (color, taste, and so on) and a strong purchase decision^11^. Drying methods are mainly composed of ND, HD, VD, fluidized bed drying, ID, MD, and FD based on different drying mechanisms^12,13^. For the reason that drying is a complex process involving continuous heat and mass transfer, it may accompany with significant changes in the nutrients, phytochemicals compositions, surface morphology and internal structure as well as physical properties of the products^8,9,14–16^, and even influence *in vitro* digestibility of substances^17^. For dried agricultural products, color is one of the most important quality indexes that influenced consumer appetite, acceptance and commodity value of products. The physical characteristics such as firmness, crispness, shrinking, and dehydration capacity are usually used to evaluate the mechanical properties and are defined as the resistance of a material to deformation or penetration, and are influenced by surface and internal structure. Most importantly, these parameters can affect the packaging, transportation and edible quality of dried products. In addition, free amino acids are important taste active compounds in edible fungi, especially Asp and Glu, which are responsible for umami flavour of edible fungi^9^. Therefore, the choice of the drying methods and processing parameters is of great importance for *Pleurotus eryngii*. Chen *et al*. only optimized the technology parameters of *Pleurotus eryngii* by microwave-vacuum drying based on quality and energy consumption^18^. Su *et al*. investigated drying characteristics and water dynamics during microwave hot-air flow rolling drying of *Pleurotus eryngii*^19^. Li *et al*. mainly studied the effects of different drying methods on the tasty compounds but did not investigate the changes in color, physical and texture properties of dried *P. eryngii*^20^. Therefore, these studies are still inadequate on the choice of drying methods, drying temperatures as well as research content and parameters for drying processing of *P. eryngii*, which can not be fully and objectively reflected the effect of different drying methods on sensory, physical and chemical qualities of dried *P. eryngii*.

The purpose of this study is to assess the influences of different drying methods and temperatures on the physicochemical properties, microstructures, nutritional compositions, and free amino acids of *P. eryngii*, and recommend a desirable drying method. These will provide valuable information to maximally retain the original nutrients and sensory qualities of *P. eryngii* during drying process.

## Results and Discussion

### Color determination

Generally speaking, the smaller the total color difference (ΔE), the closer the color to the fresh sample. The color values of fresh and dried *P. eryngii* slices dried with different methods are shown in Table 1. Except FD, all dried samples exhibited lower lightness (L* value), higher redness (a* value) and yellowness (b* value) compared with fresh *P. eryngii*, and a significant difference (*p* < 0.05) was found in L*, a*, and b* values among different drying methods. Similar results were also reported in shiitake mushrooms^9,21,22^. As expected, the L* values closest to the fresh sample and lowest ΔE values were found in FD samples (Table 1), indicating that the color parameters of FD were close to those of fresh sample. This can be explained by the fact that less oxygen was present in a drying chamber under low temperature and pressure, which led to less intense enzymatic browning reactions and non-enzymatic browning reactions. HD sample at 40 °C (HD40) possessed higher L* and lower ΔE values compared with HD at 60 °C (HD60), VD at 40 °C (VD40), VD at 60 °C (VD60), ID at 40 °C (ID40), ID at 60 °C (ID60) samples, while MD had the lowest L* and the highest ΔE values. In fact, during VD process, less oxygen was present in a drying chamber under low pressure. Hence, the principal cause of color darkening of dried *P. eryngii* could be due to the heating during drying, causing non-enzymatic browning reactions that depended on heating temperature and time. The change in color of MD *P. eryngii* slices indicated that browning occurred more intensely because of non-enzymatic browning. Similarly, some studies reported that ID shiitake mushroom, carrot and garlic exhibited better color than MD products^14,23^. The above results indicated that FD can better retain the color of dried *P. eryngii* slices.

**Table 1:** Color of fresh and dried *P. eryngii* slices.

	Color parameters			
	L*	a*	b*	ΔE
Fresh	72.8 ± 3.9ab	−0.2 ± 0.2d	4.6 ± 0.5c	
ND	55.5 ± 4.0cd	1.7 ± 0.3b	10.3 ± 0.9bc	21.3 ± 2.6 bc
HD40	64.1 ± 2.8bc	1.6 ± 0.4bc	15.5 ± 4.9b	19.4 ± 1.1 c
HD60	54.5 ± 4.2cd	1.4 ± 0.3bc	13.4 ± 2.4b	23.7 ± 2.3 bc
VD40	53.4 ± 3.7de	2.5 ± 0.4b	11.4 ± 1.2b	23.8 ± 1.3 bc
VD60	52.3 ± 2.8de	2.2 ± 0.8b	10.6 ± 1.1b	24.4 ± 1.5 bc
ID40	53.7 ± 2.4de	1.5 ± 0.4bc	11.7 ± 1.9b	23.5 ± 1.8 bc
ID60	51.8 ± 2.4de	2.4 ± 0.2b	13.2 ± 0.5b	25.8 ± 2.1 b
MD	44.6 ± 3.0e	7.9 ± 0.8a	25.8 ± 1.5a	39.3 ± 3.2 a
FD	79.3 ± 1.6a	0.4 ± 0.3cd	10.1 ± 1.9bc	11.2 ± 0.8 d

Values are expressed as mean ± SD of triplicate measurements. Different letters within a column indicate statistically significant differences between the means (p < 0.05) for L*, a*, b*and ΔE.

### Shrinkage ratio

**Figure 1:**
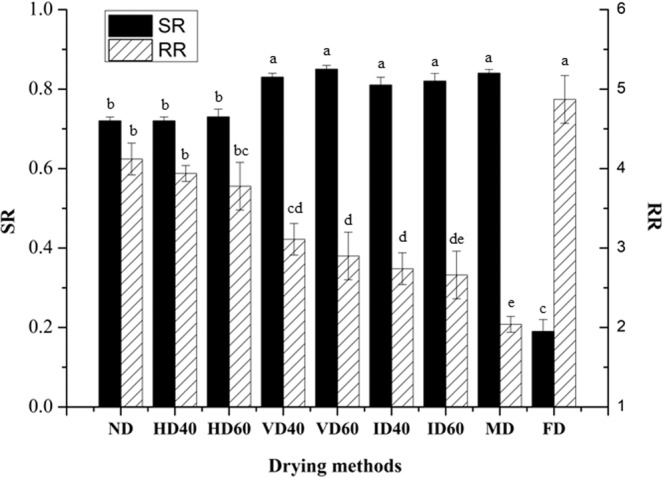
Shrinkage ratio (SR) and rehydration ratio (RR) of dried *P. eryngii* using different drying methods. Different small letters indicate statistically significant differences between the means (*p* < 0.05) for SR and RR.

The SR of different dried *P. eryngii* slices is shown in Fig. 1. VD40, VD60, ID40, ID60 and MD samples had the higher SR ranged from 0.81 to 0.85, but there was no significant difference. The followings were ND, HD40 and HD60 samples with the SR values ranged from 0.72 to 0.73. The lowest SR was found in FD products (0.19), indicating that the volume of *P. eryngii* slices remained unchanged during FD process. Drying process accompanied with a complicated mass, heat, and momentum transportation within the food products with multiple phases such as solids, liquid water, and gas^24^. In FD system, an extensive pore network was left by the sublimation of ice, and therefore, little shrinkage took place^25^. Similar to ND, HD was a convective drying process, and the shrinkage was more obvious compared to FD^14^. VD process in this study took longer time than HD under the same temperature, which made the product shrink and formed hardened crust. In ID process, the infrared radiation penetrated the product, and then converted to thermal energy through molecular vibration, and these heat was finally absorbed by the materials without heating the surrounding air^15,26,27^. For MD sample, the microwave energy created a very porous structure of the mushrooms, facilitating the transport of water vapor^24^, which resulted in cell swelling because water evaporation was accelerated by the preferential absorption of microwave energy. However, negative pressure occurred within the mushrooms, offsetting the puffing process. Hence, the SR of VD, MD and ID samples were the highest. Different results are also reported by other researchers. Baysal *et al*. reported that SR of MD sample was significantly higher than that of HD and ID samples^23^, while Tian *et al*. reported that SR of HD sample was significantly higher than that of VD and MD samples^9^. In general, VD, ID and MD mushrooms were significantly greater than that of ND and HD mushrooms (*p* < 0.05) in terms of SR, while the lowest SR was found in FD products.

### Rehydration ratio

The RR of dried *P. eryngii* slices treated with different drying methods are given in Fig. 1. FD sample showed the highest RR (4.87), followed by ND, HD40 and HD60 mushrooms, which had no significant difference (*p* < 0.05). The physical and chemical changes during drying process had significant influences on RR, so did the porosity of surface pores and internal structure of the products^14^. The ND, conventional HD and VD resulted in obvious shrinking of mushrooms, forming a dense structure, and maybe just the collapsed capillaries reduce the water retention ability during rehydration^14^. Contrary to the results of SR, the RR of MD sample was the lowest (2.04), followed by VD 60, ID 40 and ID 60 samples. Similar results were reported by some studies^14,28^. In fact, we also found the surface of MD product exhibited serious shrinkage and a hard crust, decreasing the rehydration capacity. There had little effect of drying temperature on rehydration capacity of the *P. eryngii* slices. However, samples dried by lower temperature had a relatively higher RR than those dried by higher temperature. Similar tendency was also observed in the some studies^29,30^. This may be associated with more intense moisture change of the samples at higher temperature, which caused more seriously damage to the capillaries and further resulted in modifications of osmotic properties of the cell as well as lower diffusion of water through the surface during rehydration^31^. In addition, there was moderate negative correlation (*p* < 0.05) between the RR and SR, and the correlation coefficient was 0.69 (not shown).

### Firmness and crispness

**Figure 2:**
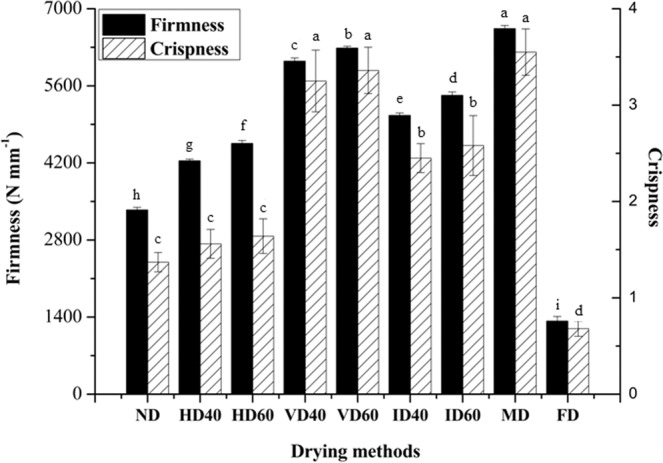
Firmness and crispness of dried *P. eryngii* using different drying methods. Different small letters indicate statistically significant differences between the means (*p* < 0.05) for firmness and crispness.

The firmness and crispness of *P. eryngii* slices dried with different drying methods are illustrated in Fig. 2. Firmness can be related to the force performed by mastication that took part during eating. The higher the firmness value, the harder it was to chew^14^. The highest firmness was observed for MD sample (6635 N mm^−1^), followed by VD60, VD40, ID60, ID40, HD60, HD40, ND, and FD samples. According to Fig. 2, MD and VD samples exhibited the highest crispness values, followed by ID samples. FD showed the lowest crispness value, followed by ND and HD. Besides, it can be observed that drying temperature had a positive effect on the firmness while no influence on the crispness. These textural variations could be explained by changes in the plant cell wall, which occurred during drying processing with different temperatures and methods, causing significant decrease in internal pressure^8,31^. Furthermore, there was a high positive correlation (*p* < 0.01) between the firmness and the crispness of dried *P. eryngii* slices, and the correlation coefficient was 0.95 (not shown).

### Microstructure

**Figure 3:**
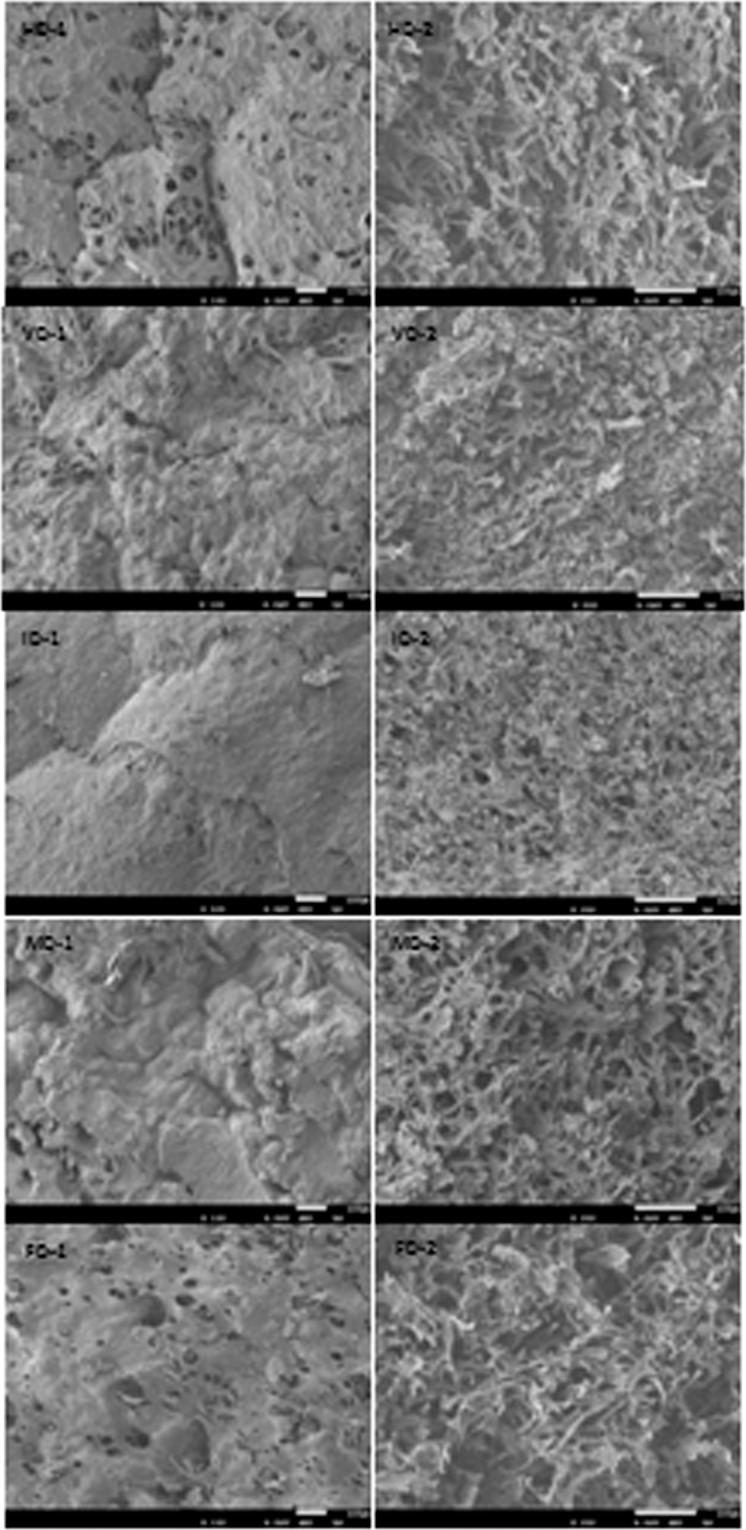
SEM micrographs of dried *P. eryngii* samples. the left, samples surface; the right, samples lateral section.

Figure 3 showed scanning electron microscopy (SEM) micrographs of the surface and lateral section of dried *P. eryngii* slices with different drying methods. The images showed that drying methods had a obvious effect on the tissue structure of *P. eryngii* slices. As expected, the FD samples showed the most plenty of pores in the surface and uniform honeycomb networks in the interior, which almost had no collapsed structure. This phenomenon can be explained by the fact thatthe original dimensions of the product were maintained first by freezing during FD process, then the ice was sublimed under a high vacuum, and thus the destruction of FD products cells could be minimized^32^. It also provided a good way for the infiltration of water during rehydration process, and thus the highest RR, the lowest SR. Compared with FD sample, larger but less pores were seen in the surface of HD samples which indicated more cellular tissue collapse and shrinkage. The morphology in the interior of the HD samples was relatively dense, which partly explained the increasing firmness in HD samples. Compared with HD, VD samples had an irregular surface with a few small cavities, while the organizational structure revealed multiple channels and large irregularities. In VD process, the drying process was conducted under negative vacuum pressure, and thus a reducing boiling point of water made the whole process more rapidly. Finally, a very porous interior structure was formed in the interior of VD samples^24^. A long time high temperature treatment may cause a collapsed structure, a rapid shrinkage and a hard shell, all of which affected the water absorption capacity of the materials^33^. ID samples showed a dense surface layer or crust with few holes but a porous and densest internal structure, which may be due to the limited depth of infrared radiation when penetrating from the surface into the interior. The uneven distribution of water diffusion as well as the collapse of the cellular structure on the surface finally resulted in the uneven structures in ID samples^14^. Different results were reported by Pan *et al*. who demonstrated that application of ID was better compared to HD for the microstructure of dehydrated banana^34^. The surface of MD samples stacked together and showed almost invisible pores but had a clear porous internal structure. Microwave energy had the ability of selective heating on the interior portions of the products and created a high steam pressure. The dried surface looks like a barrier to hinder the release of the pressure and the product was thus puffed^8^, which also led to a hardening texture and a poor rehydration property. Besides, a lot of soluble viscous polysaccharide substances were transported to the surface when ID and MD were applied to *P. eryngii*. In consequence, a hard crust would be formed in both ID and MD products, just like roasting, and these samples also got the lowest RR and highest firmness and crispness values.

### Nutrient components

The chemical compositions of fresh and dried *P. eryngii* slices treated with different drying methods are listed in Table 2. The moisture content of fresh *P. eryngii* was 89.8% based on fresh weight (fw). Largely consistent with the previous results^35^, other proximate compositions are listed in the following order (g/100 g dry weight (dw)): carbohydrate (63.4 ± 0.5) > protein (22.1 ± 0.2) > ash (6.5 ± 0.2) > reducing sugar (2.9 ± 0.3) > fat (2.5 ± 0.2). As expected, the final moisture contents of dried *P. eryngii* using different drying methods ranged from 4.2 to 4.9 g/100 g dw and showed no significant difference (p < 0.05).

**Table 2:** Moisture content and proximate compositions (g/100 g dw) of fresh and dried *P. eryngii* samples.

	Moisture	Protein	Fat	Ash	Reducing sugar	Carbohydrate
Fresh		22.1 ± 0.2b	2.5 ± 0.2a	6.5 ± 0.2a	2.9 ± 0.3a	63.4 ± 0.5e
ND	4.7 ± 0.4a	21.9 ± 0.1b	2.3 ± 0.1a	6.7 ± 0.3a	2.8 ± 0.2a	63.3 ± 0.7e
HD40	4.6 ± 0.3a	21.4 ± 0.3bc	2.3 ± 0.3a	6.4 ± 0.1a	2.4 ± 0.1ab	64.3 ± 0.5e
HD60	4.5 ± 0.2a	20.8 ± 0.4c	2.4 ± 0.1a	6.3 ± 0.4a	2.0 ± 0.2bc	65.0 ± 0.6de
VD40	4.9 ± 0.5a	18.2 ± 0.2d	2.3 ± 0.2a	6.5 ± 0.5a	2.0 ± 0.2bc	67.1 ± 0.4 cd
VD60	4.8 ± 0.3a	17.5 ± 0.5de	2.2 ± 0.4a	6.4 ± 0.6a	1.5 ± 0.1cd	68.1 ± 0.5bc
ID40	4.5 ± 0.4a	17.9 ± 0.1de	1.5 ± 0.2b	6.6 ± 0.3a	1.9 ± 0.2bc	68.5 ± 0.6bc
ID60	4.4 ± 0.2a	17.1 ± 0.4e	1.4 ± 0.1b	6.4 ± 0.2a	1.3 ± 0.1de	69.7 ± 1.3ab
MD	4.2 ± 0.4a	15.6 ± 0.3f	1.2 ± 0.3b	6.9 ± 0.6a	0.8 ± 0.1e	71.1 ± 1.0a
FD	4.4 ± 0.6a	25.7 ± 0.2a	2.1 ± 0.2a	6.5 ± 0.4a	2.9 ± 0.3a	60.3 ± 0.8 f

Values are expressed as mean ± SD of triplicate measurements. Different letters within a column indicate statistically significant differences between the means (p < 0.05) for moisture, protein, fat, ash, reducing sugar and carbohydrate.

The results revealed that the ash contents, ranging from 6.3 to 6.9 g/100 g dw, were not affected by drying methods. The protein contents of samples using different drying methods are listed in the following order: FD > fresh, ND and HD40 > HD60 > VD40, VD60 and ID40 > ID60 > MD. There was no significant difference in fat content among the fresh, FD, ND, HD, and VD samples, while ID and MD showed significant lower fat contents. During ID, the rapid heating of mushrooms by infrared radiation may promote fat oxidation^15^. Unlike ID, heating was generated within the samples in MD, which greatly improved the heating efficiency and also caused fat oxidation. The reducing sugar contents of samples using different drying methods are listed in the following order: fresh, FD, ND and HD40 > HD60, VD40, VD60 and ID40 > ID60 and MD. Contrary to fat content, MD and ID 60 samples got the highest values in carbohydrate contents. The chemical compositions of FD, ND and HD40 samples showed high similarity to fresh *P. eryngii*.

### Free amino acids

The contents of free amino acids (FAA) compositions of fresh and dried *P. eryngii* slices treated with different drying methods are given in Table 3. Glutamic acid (Glu), alanine (Ala), phenylalanine (Phe) and lysine (Lys) exhibited higher amounts in all *P. eryngii* samples, which was similar to the previous reports^36–38^. The content of total FAA (TFAA) in fresh sample was 59.06 mg/g dw, which was much higher than the previous report with 18.07 mg/g dw of TFAA^20^. The TFAA content decreased significantly (*p* < 0.05) in the dried samples with a descending order as follows: FD > ND > HD40 > VD40 > HD60 > ID40 > VD60 > ID60 > MD. It was worth mentioning that the FD product contained more Asp, Phe, Tyr and Ser than the fresh mushroom. During FD process, some free amino acids including Glu, His, and Phe, might be released during proteolysis, and after that, the contents of free amino acids decreased due to the strecker degradation of free amino acids and the Maillard reaction^39–42^. Eight essential amino acids (EAA) for humans were detected in these samples, and the total contents of EAA (TEAA) ranged from 5.03 to 26.31 mg/g. The lowest concentration was observed in MD sample and the highest in fresh sample. The contents of non-essential amino acids (NEAA) of samples had the same trend with EAA and TFAA. The contribution of total free amino acids to the total crude protein was calculated and showed in Table 3. The percentage of TFAA/protein in different samples ordered similar to the content of TFAA. Heating would promote proteolysis as well as non-enzymatic Maillard reactions between amino acids and reducing sugars in mushrooms during the drying process^39^. These results agreed with those for protein content, reducing sugar content and color values, suggesting the amount of FAA released from proteolysis was lower than that of FAA lost in Maillard reaction during ND, HD, VD, ID and MD drying process, and the higher temperature can led to mushrooms darken and hardness^12^.

**Table 3:** Contents of free amino acids of fresh and dried *P. eryngii* samples.

Amino acids	Fresh	ND	HD40	HD60	VD40	VD60	ID40	ID60	MD	FD
				Amino acid content (mg/g)						
Glycine	2.12 ± 0.15	1.71 ± 0.21	1.33 ± 0.16	1.07 ± 0.11	1.75 ± 0.26	1.32 ± 0.16	1.44 ± 0.06	1.23 ± 0.66	0.14 ± 0.08	2.05 ± 0.16
Alanine	6.44 ± 0.36	5.29 ± 0.32	4.68 ± 0.53	3.31 ± 0.23	3.48 ± 0.52	2.55 ± 0.21	2.94 ± 0.35	2.16 ± 1.38	1.45 ± 0.20	5.83 ± 0.34
Valine	0.74 ± 0.11	0.06 ± 0.01	nd	nd	nd	nd	nd	nd	nd	0.42 ± 0.33
Isoleucine	2.55 ± 0.24	1.89 ± 0.61	1.36 ± 0.08	1.05 ± 0.15	1.39 ± 0.14	0.88 ± 0.09	0.31 ± 0.04	0.14 ± 0.03	0.26 ± 0.14	2.14 ± 0.21
Leucine	4.32 ± 0.20	3.79 ± 0.22	4.08 ± 0.37	4.84 ± 0.29	3.92 ± 0.41	3.73 ± 0.24	3.25 ± 0.53	2.66 ± 0.19	1.46 ± 0.16	4.03 ± 0.28
Aspartic acid	3.30 ± 0.25	1.52 ± 0.12	1.55 ± 0.19	1.38 ± 0.11	1.47 ± 0.22	1.15 ± 0.15	1.22 ± 0.21	0.95 ± 0.11	0.34 ± 0.07	3.55 ± 0.33
Glutamic acid	8.25 ± 0.54	5.78 ± 0.33	5.56 ± 0.41	6.25 ± 0.38	4.64 ± 0.21	3.99 ± 0.30	4.77 ± 0.35	4.43 ± 0.38	2.12 ± 0.15	7.60 ± 0.58
Arginine	1.48 ± 0.26	0.51 ± 0.06	0.31 ± 0.22	0.11 ± 0.02	0.45 ± 0.07	0.08 ± 0.01	0.05 ± 0.01	nd	nd	1.33 ± 0.09
Lysine	5.33 ± 0.52	4.94 ± 0.15	3.12 ± 0.29	2.08 ± 0.15	3.43 ± 0.17	1.04 ± 0.16	3.54 ± 0.39	1.24 ± 0.99	0.75 ± 0.09	4.24 ± 0.22
Histidine	2.47 ± 0.35	1.33 ± 0.07	1.10 ± 0.07	0.75 ± 0.04	0.50 ± 0.06	0.44 ± 0.05	1.12 ± 0.31	0.56 ± 0.36	0.42 ± 0.05	1.97 ± 0.21
Phenylalanine	4.96 ± 0.36	4.13 ± 0.20	4.00 ± 0.36	3.20 ± 0.14	4.34 ± 0.52	4.05 ± 0.28	3.06 ± 0.42	2.75 ± 0.17	1.55 ± 0.13	5.11 ± 0.42
Tyrosine	2.98 ± 0.21	2.54 ± 0.36	2.13 ± 0.34	1.78 ± 0.32	2.34 ± 0.21	1.90 ± 0.20	1.77 ± 0.14	1.12 ± 0.08	0.54 ± 0.04	3.54 ± 0.13
Threonine	3.32 ± 0.31	2.82 ± 0.09	2.22 ± 0.16	1.45 ± 0.24	1.56 ± 0.08	1.13 ± 0.14	0.34 ± 0.44	0.88 ± 0.11	1.02 ± 0.06	3.21 ± 0.36
Serine	1.93 ± 0.26	1.75 ± 0.35	1.15 ± 0.26	1.82 ± 0.14	1.06 ± 0.09	0.48 ± 0.06	0.78 ± 0.62	0.55 ± 0.06	0.83 ± 0.05	2.22 ± 0.17
Tryptophan	4.35 ± 0.41	2.90 ± 0.31	1.70 ± 0.14	0.96 ± 0.13	1.54 ± 0.27	nd	nd	nd	nd	3.54 ± 0.24
Cysteine	0.85 ± 0.13	0.24 ± 0.03	nd	nd	nd	nd	nd	nd	nd	0.50 ± 0.05
Methionine	0.74 ± 0.10	0.58 ± 0.06	0.15 ± 0.03	nd	0.06 ± 0.01	nd	nd	nd	nd	0.64 ± 0.08
Proline	2.93 ± 0.31	1.82 ± 0.08	1.46 ± 0.15	0.69 ± 0.10	0.94 ± 0.13	0.14 ± 0.06	0.07 ± 0.01	0.08 ± 0.01	nd	1.88 ± 0.11
TEAA	26.31 ± 1.62	21.11 ± 3.55	16.63 ± 0.13	13.58 ± 1.56	16.24 ± 2.30	10.83 ± 0.89	10.50 ± 0.69	7.67 ± 0.64	5.04 ± 0.48	23.33 ± 1.36
NEAA	32.75 ± 0.25	22.49 ± 0.34	19.27 ± 0.61	17.16 ± 0.99	16.63 ± 1.88	12.05 ± 0.93	14.16 ± 1.57	11.08 ± 1.12	5.84 ± 0.35	30.47 ± 2.06
TFAA	59.06 ± 3.23	43.60 ± 0.28	35.90 ± 2.78	30.74 ± 2.33	32.87 ± 2.06	22.88 ± 1.57	24.66 ± 1.98	18.75 ± 1.38	10.88 ± 0.92	53.80 ± 3.27
				TFAA/protein (%)						
	26.72 ± 2.12	19.91 ± 1.76	16.78 ± 2.01	14.78 ± 1.64	18.06 ± 2.03	13.07 ± 1.63	13.78 ± 1.20	10.96 ± 0.88	6.97 ± 0.45	20.93 ± 1.66

nd, not detected. Values are expressed as mean ± SD of triplicate measurements.

### Differentiation by cluster analysis

**Figure 4:**
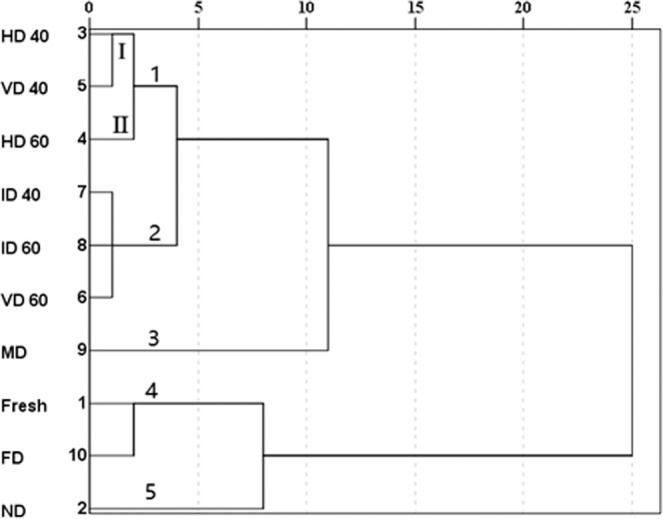
Clustering results of FAA data analysis of fresh and dried *P. eryngii* using different drying methods.

In order to establish differences among different drying methods, a cluster analysis is conducted on FAA contents according to squared Euclidean distance methods (Fig. 4). The results showed that all samples were divided in to five different clusters. Cluster 1 included three drying samples (HD40, VD40 and HD60), which was further divided into two sub-clusters (I and II). The TEAA contents of HD40 and VD40 samples in sub-cluster I showed high similarity due to the same temperature, which was the critical factor of Maillard reaction causing the loss of FAA, while HD60 sample in sub-cluster II was different. The TFAA content of VD40 sample was lower than that of HD40, due to the longer drying time in VD process. ID40, ID60 and VD60 products were brought together in cluster 2. With the Euclidean distance increasing, cluster 1 and 2 got together, which meant that there was a similarity between two clusters. Cluster 3 was grouped with others, which appeared the sample dried by MD with low similarity with others. Fresh sample in cluster 4 exhibited the highest TFAA and high similarity to FD sample, which meant FD process was the most effective drying method to preserve the FAA of *P. eryngii*. The cluster 5 contained only ND sample. Besides, as the Euclidean distance increased, clusters 4 and 5 got together, which meant that ND sample had more similarities with fresh and FD samples than others.

## Conclusion

In conclusion, the physicochemical properties, microstructures, nutritional compositions, and free amino acids of *Pleurotus eryngii* were significantly affected by different drying methods. Among them, FD was the most effective drying method and retained the main characteristics of the fresh *P. eryngii* in physicochemical properties, microstructures, nutritional compositions and free amino acids, followed by ND and HD40. However, it was worth mentioning that FD equipments was rather expensive, and the processing procedure was time and energy consuming. MD treatment damaged the physicochemical properties of *P. eryngii* slices and resulted in massive losses of the main nutrients and free amino acids. These results will provide a theoretical basis for industrial processing of *P. eryngii*. It is well known that drying must have an influence of on the bioactivity of the substrate. Therefore, the effects of drying methods on bioactive substances including active polysaccharides and phenols and biological activities of *P. eryngii* still need to be further studied. In addition, further studies need to be performed on the drying process to improve the nutritional quality and health-caring quality of *P. eryngii*.

## Materials and Methods

### Materials preparation

Fully mature fresh *P. eryngii* that is called Xingbaogu in China was harvested from Aokun Biological Agriculture Co. Ltd., Shanxi Province, China. Prior to drying, the fresh *P. eryngii* samples were cut into slices with thickness of 5.0 ± 0.2 mm, and then were divided into ten portions at random. Among them, one was used for fresh analysis, and other batches were respectively dried with different methods in optimized conditions to get a water content ( ≤ 5% dry base). The moisture content of fresh *P. eryngii* was 89.8 ± 1.7% (wet basis), which determined with a hot air oven at 105 °C. After cutting, slices were processed immediately.

### Drying process

The *P. eryngii* slices were dried using the following treatments (1) ND was carried out under natural air flow and ambient temperatures (23–30 °C) for 4 days, (2) HD was conducted in a draught drying cabinet (GZX-9246MBE, Yueming Scientific Instrument Ltd., Shanghai, China) at 40 °C for 13 h or 60 °C for 8 h until constant weight was achieved respectively, (3) VD was realized with a vacuum drier (DZF-6090, Hecheng Instrument Manufacturing Co., Ltd., Shanghai, China), and the samples were dried at 40 °C for 19 h or 60 °C for 12 h with vacuum of 0.08 MPa, (4) ID was performed in a laboratory scale benchtop infrared drier (YHG 600BS, Botai Laboratory Equipment Ltd., Shanghai, China), and the samples were dried at 40 °C for 14 h or 60 °C for 6 h, (5) MD was done in a microwave oven (WD900Y, Galanz Enterprise Group Co. Ltd., Guangdong, China) at 240 W for 10 min, (6) FD was carried out in a freeze drier at 20 °C and 30 Pa for 24 h.

### Color measurements

The CIE color coordinates (L*, a*, b*) of fresh and dried *P. eryngii* slices were determined with a CM-700D colorimeter (Konica Minolta Optics Inc., Japan). The ΔE was calculated according to the formula^21^,

$$\Delta E=\sqrt{{({{\rm{L}}}^{\ast }-{{\rm{L}}}_{0})}^{2}+{({{\rm{a}}}^{\ast }-{{\rm{a}}}_{0})}^{2}+{({{\rm{b}}}^{\ast }-{{\rm{b}}}_{0})}^{2}},$$

where L_0_, a_0_, and b_0_ referred to the color reading of fresh samples, which was used as control.

### Shrinkage ratio

The shrinkage ratio (SR) was measured by a displacement method^14^. The shrinkage ratio of dried *P. eryngii* slices was calculated according to the formula, SR = (V_1_ − V_2_)/V_1_, where V_1_ and V_2_ were the volume (cm^3^) of the fresh and dried samples, respectively.

### Rehydration ratio

The rehydration ratio (RR) was determined according to the reported method^43^. Dried slices were weighed (W_0_) and immersed for 30 min in distilled water at room temperature. Then the rehydrated samples were removed, and the slices were weighed again (W_1_). The RR was calculated according to the formula, RR = (W_1_ − W_0_)/W_0_.

### Firmness and crispness

Firmness and crispness of dried *P. eryngii* slices were evaluated by means of a puncture test carried out by a Texture Analyzer. The probe used was cylindrical with a puncture diameter of 10 mm and the parameters were preset as follows: test speed 1.0 mm s^−1^, pretest speed 3.0 mm s^−1^, posttest speed 3.0 mm s^−1^, travel distance of 3.0 mm. Firmness (N mm^−1^) of samples was defined as the maximum force applied to puncture the mushroom. The crispness was defined as the coordinate of the first peak pressure.

### Microstructure analysis

The surface morphology and internal structure of dried *P. eryngii* were observed by scanning electron microscopy (Model JSM-5310LV; JEOL Ltd., Tokyo, Japan) at an accelerating voltage of 6.0 kV. The SEM micrographs of the surface and interior of samples were obtained at 100 × magnification and 200 × magnification respectively.

### Nutrient components analysis

Ash, protein and fat were determined following the AOAC procedures^44^. The nitrogen factor used for crude protein calculation was 4.38^13^. Fat percentage was determined using a Soxhlet apparatus. The content of reducing sugar was estimated by the 3, 5-dinitrosalicylic acid spectrophotometric method^10,16^.

### Free amino acids analysis

Free amino acid extraction and analysis were carried out according to the method reported by Li *et al*.^16^. The 0.2 g powder samples were added to 20 mL 75% ethanol and shaken with a laboratory rotary shaker at 150 rpm for 30 min at 70 °C. After centrifugation at 10000 g and 4 °C for 15 min, the supernatant was collected, evaporated to dryness. The residual was reconstituted with pH 2.2, 0.2 M sodium citrate loading buffer solution to a final volume of 10 mL. The standard solution and prepared filtrate were analyzed by a automatic amino-acid analyzer (Biochrom Ltd., England). The injection volume and the duration of single run were 20 µL and 60 min, respectively. The amino acids were identified and quantified by comparing peak profiles of the mushroom samples with standard amino acid profiles.

### Statistical analysis

All experiments were carried out in triplicate and the data were expressed as mean ± standard deviation (SD). Correlation coefficient, One-way analysis of variance (ANOVA) and Duncan’s multiple range test were carried out to determine significant differences (*p* < 0.05) between the means.
